# Impact of frailty and mobility on mortality and symptomatic improvement in TAVR

**DOI:** 10.1016/j.ijcha.2025.101725

**Published:** 2025-06-19

**Authors:** Samer Fawaz, Christopher Cook, Ashni Chani, Sarosh Khan, Sanjay Rajput, John Davies, Thomas R Keeble, Rajesh Aggarwal, Rohan Jagathesan, Ozan M. Demir

**Affiliations:** a Essex Cardiothoracic Centre, Mid and South Essex NHS Foundation Trust, Basildon, UK; b Anglia Ruskin School of Medicine & MTRC, Anglia Ruskin University, Chelmsford, UK; c University College London, London, UK

**Keywords:** Aortic stenosis, Frailty, Mobility, Prognosis, TAVR, TAVI

## Abstract

• Frailty and poor mobility predict higher 1-year mortality and reduced symptomatic improvement after TAVI.
• Diabetes, chronic kidney disease, and male sex are linked to increased 1-year mortality.
• Respiratory disease and female sex are associated with less symptomatic benefit post-TAVI.

Transcatheter aortic valve replacement (TAVR) is an established treatment for severe aortic stenosis [1]. Despite iterative procedural improvements, variability in patient outcomes underscores the need for enhanced predictors of post-TAVR recovery and mortality, with frailty and mobility emerging as potential factors influencing prognosis [2]. Furthermore, recent studies have demonstrated the benefits of TAVR in asymptomatic patients, highlighting the importance of careful patient selection [3]. Accordingly, we evaluated the influence of frailty and mobility scores in a contemporary TAVR population by investigating their impact on: (a) symptomatic improvement at 6–8 weeks post-TAVR; and (b) 1-year post-TAVR mortality.

Consecutive patients who underwent TAVR at the Essex Cardiothoracic Centre, United Kingdom, between 2017 and 2023 were included in the study. All clinical data was prospectively collected. Symptomatic status was reviewed 6–8 weeks post-TAVR through outpatient clinic assessment. Patients' survival data were obtained by linkage of patients' National Health Service numbers to the Office of National Statistics. The UK National Research Ethics Service approved the study (REC 24/HRA/4695).

Symptomatic improvement was assessed using a patient-reported scale, ranging from −3 (“much worse”) to + 3 (“much better”). One-year mortality was assessed with Kaplan-Meier survival curves, followed by Cox-regression hazards modelling to evaluate the impact of frailty and mobility on survival. Frailty was graded using the Canadian Study of Health and Aging (CSHA) frailty score, while mobility was classified as “poor” if the 4-meter walk test speed was < 0.8 m/s. Multivariate linear regression analysis was conducted investigating the relationship between frailty, mobility, and symptom improvement, incorporating additional factors such as respiratory disease, chronic kidney disease (CKD), gender, and age. Due to limited sample size, frailty scores of 1, 2, and 6 were excluded from the analysis, representing a total of seven patients. Statistical analyses were performed using SPSS (version 29) and R Studio (version 2024.09.1).

The study population consisted of 651 consecutive patients with a mean age 82.1 ± 5.5 years, 41.2 % females, 28.3 % diabetes mellitus and mean left ventricular function 52 ± 10 %. Most patients (81.3 %) were CSHA grade 4, indicating mild frailty, while smaller groups were CSHA 3 (11.4 %) and CSHA 5 (7.4 %). There was no difference in mean aortic valve gradient, diabetes mellitus, respiratory disease, and CKD between CSHA groups (ANOVA p > 0.05 for all). A small age difference was observed between CSHA groups (CSHA 3: 80 ± 6 years; CSHA 4: 82 ± 5 years; CSHA 5: 82 ± 6 years; p < 0.001).

Symptomatic improvement post-TAVR was inversely associated with CSHA grade, with higher frailty scores linked to significantly lower symptom improvements (mean symptom improvement scores: 2.6 ± 0.8, 1.9 ± 0.1, and 1.2 ± 0.3 for CSHA 3, 4, and 5, respectively; ANOVA p < 0.001) (Fig. 1). Furthermore, poor mobility was negatively associated with symptomatic recovery (1.4 ± 0.2 vs 2.1 ± 0.1; p < 0.001). Multivariate linear regression identified frailty, poor mobility, respiratory disease, and female gender as predictors of poor symptomatic improvement following TAVR (p < 0.05 for all).

**Fig. 1:**
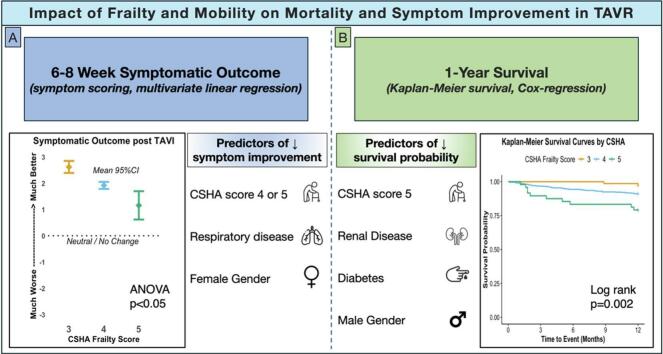
Central Illustration. Symptom score by CSHA frailty score, and predictors of reduced symptom improvement (panel A); 1 year Kaplan-Meier survival curves by CSHA frailty score, and predictors of reduced survival probability (Panel B).

Kaplan-Meier survival curves showed a significant 1-year mortality difference by CSHA score (log-rank p = 0.002) (Figure 1B). Multivariable Cox-regression revealed that frailty (CSHA 5 compared to CSHA 3: HR = 5.31 [95 % CI 1.07–26.28; p = 0.04]) and poor mobility (HR = 2.13 [95 % CI 1.12–4.05; p = 0.02]) were significant predictors of post-TAVR mortality. Additionally, diabetes, CKD, and male sex were independent predictors of increased mortality (p < 0.05 for all). Importantly, these factors affected mortality independently of age (HR 1.01; 95 % CI 0.96–1.05; p = 0.66), indicating that frailty and mobility may have a greater impact on post-TAVR survival than chronological age.

There are limitations to this study. First, this was a retrospective analysis of prospectively collected data used for other purposes, and although the data were of high quality, it is not possible to rule out inherent biases from retrospective observational dataset analysis. Second, there were limited numbers of patients within the CSHA 3 and 5 groups rendering propensity matching not feasible without sacrificing the overall sample size. Lastly, although a clear difference in symptom outcome was noted using our scoring system, conventional symptom and quality of life scoring systems were not utilized, as this is not routine clinical practice in our center.

This study demonstrates that frailty and mobility are significant predictors of symptomatic improvement at 6–8 weeks and 1-year mortality in a contemporary TAVR cohort. As indications for TAVR potentially expand to include asymptomatic patients with severe aortic stenosis, meticulous patient selection emerges as an increasingly critical component of the pre-procedural evaluation process [3]. Furthermore, our study highlights notable differences in outcomes based on gender, with women demonstrating improved survival yet experiencing comparatively less symptomatic benefit post-TAVR. This aligns with prior literature suggesting sex-based differences in recovery trajectories following valve interventions [4]. Although frailty has previously been well-established as a prognostic indicator in TAVR populations, our findings uniquely contribute by simultaneously assessing short-term symptomatic improvement and long-term mortality within the same contemporary cohort. Crucially, we identified distinct sets of predictors for these outcomes beyond frailty and mobility alone. Specifically, female sex and respiratory disease were independently associated with reduced symptomatic improvement, yet they did not significantly influence mortality. Conversely, diabetes, chronic kidney disease, and male sex were significant predictors of increased mortality but did not appear to affect symptomatic improvement. These differential predictors underscore the likelihood that short-term symptom relief and longer-term survival post-TAVR represent separate physiological and pathological dimensions. Symptomatic recovery may be predominantly influenced by reversible or modifiable functional impairments and subjective patient perceptions, whereas mortality is likely driven by irreversible systemic conditions and cumulative end-organ dysfunction. Recognizing this distinction is essential for clinical decision-making and counselling, particularly as TAVR moves toward broader patient populations, including asymptomatic and lower-risk patients. Currently, no standardized TAVR risk score is routinely implemented in clinical practice. Findings from this study highlight the crucial role of comprehensive frailty and mobility assessments in the preoperative evaluation of TAVR candidates. Integrating these factors into patient selection and clinical decision-making may improve risk stratification, optimize outcomes, and inform individualized therapeutic approaches in TAVR.

## CRediT authorship contribution statement

**Samer Fawaz:** Writing – review & editing, Writing – original draft, Visualization, Methodology, Investigation, Formal analysis, Data curation, Conceptualization. **Christopher Cook:** Writing – review & editing, Supervision, Investigation, Data curation, Conceptualization. **Ashni Chani:** Writing – review & editing, Investigation, Formal analysis, Data curation. **Sarosh Khan:** Writing – review & editing, Writing – original draft, Methodology, Investigation, Formal analysis, Data curation. **Sanjay Rajput:** Writing – review & editing, Data curation. **John Davies:** Writing – review & editing, Supervision, Methodology, Conceptualization. **Thomas R Keeble:** Writing – review & editing, Methodology, Investigation, Conceptualization. **Rajesh Aggarwal:** Writing – review & editing, Supervision, Methodology, Data curation. **Rohan Jagathesan:** Writing – review & editing, Supervision, Investigation, Data curation. **Ozan M. Demir:** Writing – review & editing, Writing – original draft, Supervision, Methodology, Investigation, Formal analysis, Data curation, Conceptualization.

## Funding

None.

## Declaration of competing interest

The authors declare that they have no known competing financial interests or personal relationships that could have appeared to influence the work reported in this paper.
